# Primary health care: a necessary, current and profitable investment

**Published:** 2015-09-30

**Authors:** Sandra Girón

**Affiliations:** Escuela de Salud Pública. Editora Asociada Revista Colombia Médica. Universidad del Valle, Cali, Colombia

Traditionally, the primary health care has focused on education and health campaigns, as part of a strategy to address the diseases of interest in public health 1. As many of these problems still remain in the public health agenda, it is urgent to review the concept of primary health care. In the past, the control of vaccine-preventable diseases was its main success 2, 3. For the future, it is necessary to consider primary health care as a strategy of high impact, and to stop looking at it as a series of low-cost activities, limited to the services provided by promoters and volunteers, with low complexity technologies to serve the poor. 

The health system requires a leading primary health care, worthy to be seen as an investment with social returns, understood as the overcoming of benefits (weighted) against the costs incurred 4- 6. Thus, the primary health care strategy could be the strategy that succeeds integrating private and social interests, by achieving that the earnings of prevention contribute to the economic sustainability and the improving of health conditions for the population in general. These should be common interests to both the institutions that provide services and the health system. 

The scope of primary care leadership is not obtained without any effort. It requires investing in qualified professionals and developing interventions and innovative technologies of proven effectiveness. The costs are high and redistribution of resources will be required, which will return with results of great impact in the medium and long term to support the goals and indicators proposed in the programs. In particular, addressing infectious diseases, such as tuberculosis, in which it has become evident the need for innovation; it could greatly benefit from primary care. For example, the use of geo-referencing systems could improve the valid and timely identification of respiratory symptomatic patients and positive cases in the general population 7, 8. The implementation of home delivery systems of treatment or monitoring via video cameras have proved cost-effective in improving adherence to treatment 9, 10. By improving the timeliness of diagnosis and ensuring successful treatment, the costs of complications, the costs of drug resistance, and even the social losses due to premature death are inevitably avoided. 

Similarly, the use of technological applications such as the access to rapid diagnostic tests and the benefits of advertising on prevention have given successful results in controlling transmission and the adherence to treatment for HIV / AIDS 11- 13. 

In Colombia, a contradictory situation arises. While health is promoted as a universal right, the institutions that provide health services declare to be in economic difficulties. To keep the emphasis on essentially healing actions will result in higher costs, possibly even unsustainable costs. It is required to exit the naiveté of thinking about "saving" today's care resources, and to have an innovative approach for prevention. 

The call is to invest in research for the development of technologies and interventions that bring the health system to the population; to train professionals capable of leading a primary, innovative and sustainable care; to make use of research results in the design of programs in health, so that they integrate interventions of proven cost-effectiveness; and to formulate health policies with continuity based on primary health care, as a strategy allied to institutions and the system, in a general sense. Only that way, will the universal right to health care -enacted for so long- become real. 
